# Do players in Spanish professional soccer leagues maintain match running performance until the end of the match? A 5-minute interval analysis by match participation

**DOI:** 10.5114/biolsport.2025.151645

**Published:** 2025-08-04

**Authors:** Tomás García-Calvo, David Lobo-Triviño, José C. Ponce-Bordón, Borja Sanabria-Pino, Roberto López del Campo, Ricardo Resta, Paweł Chmura

**Affiliations:** 1 Faculty of Sport Sciences, University of Extremadura, Cáceres, Spain; 2 Department of Competitions and Mediacoach, LaLiga, Madrid, Spain; 3 Department of Individual and Team Sports, Faculty of Physical Education and Sport, Wroclaw University of Health and Sport Sciences, Wrocław, Poland

**Keywords:** Fatigue, Football, Match analysis, Physical demands, Professional

## Abstract

The present study aimed to analyse the match running performance differences across 5-minute time intervals considering the players’ participation in matches. A total of 381,194 individual match observations from professional soccer players competing in the First (n = 171,957) and Second (n = 209,237) Spanish soccer leagues over the 2022/23 season were collected. Soccer players were classified according to their participation in matches. Total distance (TD), very high-speed running (VHSR, 21–24 km × h^−1^), and sprinting speed running distance (Sprint, > 24 km × h^−1^) were analysed using a computerized tracking system (TRACAB, Chyronhego, New York, NY). Match data were divided into pre-defined 5-minute intervals. Linear mixed models were performed to analyse match running performance over the 5-minute intervals while controlling the influence of match participation. The main results showed a decrease in match running performance as the match time progressed, from the 55^th^–60^th^ minute onward (p < .001), although match participation also had a significant influence. Specifically, substitute players covered significantly greater VHSR (p < .001) and Sprint distances (p < .001) than the rest of the players during their time of participation. These findings provide knowledge about the evolution of match running performance over match time. Finally, analysis of 5-minute intervals may help coaches understand the periods of matches where player substitutions are most effective.

## INTRODUCTION

The match running performance of professional soccer players has been extensively studied over the years, revealing a dynamic alternation between low-intensity activities (e.g., walking or jogging) and high-intensity efforts (e.g., high-speed running or sprinting) [1]. Nevertheless, most match analyses have been conducted using data aggregated over the entire match, while fewer studies have focused on the physical demands from a segmented perspective, examining specific phases or periods of play [2]. Consequently, there is limited knowledge regarding the evolution of match running performance across different time intervals and how various factors influence performance during specific periods or halves of a match [3].

Professional soccer clubs and practitioners aim to enhance players’ physical reserves and pacing strategies in preparation for competition [4]. Understanding how match running performance evolves throughout a game is essential to achieve this goal. Studies have shown that soccer players’ running performance fluctuates over match time, often with a marked decline in activity during the second half [5]. For instance, Kołodziejczyk et al. [6] analysed UEFA Champions League (UCL) matches from the 2020/21 season, finding that players achieved significantly higher average speeds and covered shorter walking distances in the first half compared to the second. Furthermore, a detailed analysis of 5-minute intervals revealed distinct variations in the most demanding periods of both halves. However, these findings are not solely attributable to physiological fatigue [7] but are also influenced by game interruptions [8]. Recent research has highlighted that stoppages in play significantly affect players’ performance, leading to reductions in match running metrics [9, 10]. For example, during the group stage of the 2020/21 UCL, players exhibited a progressive decline in running performance across successive 15-minute periods, with total distance covered decreasing more substantially than high-intensity running efforts [11].

Despite the relevance of this information, none of the aforementioned studies have compared players’ match running performance across short interval periods while accounting for their match participation (i.e., starters or substitutes). To achieve a comprehensive understanding of the actual physical demands on soccer players, practitioners should consider match participation, as research has indicated that workload may be influenced by this variable [12]. Several studies have shown that substitutes exhibit higher running performance relative to playing time compared to the players they replaced and those who completed the entire match [13–16]. Specifically, these studies have demonstrated that substitutes can enhance the overall match running performance by covering greater high-intensity running distances compared to starters or replaced players [17]. Therefore, players’ different participation types during matches should be carefully considered to identify specific levels of fatigue or underperformance and implement timely tactical adjustments to influence the match outcome [18].

The literature suggests that fatigue is a key factor influencing players’ physical performance. However, the decline in soccer players’ physical performance may also be affected by contextual-related variables and tactical strategies. Consequently, the aim of this study was to analyse differences in match running performance across 5-minute intervals, taking into account players’ roles in matches. This research seeks to bridge the existing gap by providing a more detailed analysis of the evolution of match running performance across 5-minute intervals in elite soccer. The present study relies on a large dataset of matches and also examines the influence of players’ match participation. We hypothesize that the evolution of match running performance across 5-minute intervals varies significantly depending on players’ roles in the match.

## MATERIALS AND METHODS

### Study design

A retrospective, descriptive longitudinal study was conducted to analyse differences in match running performance across 5-minute periods in Spanish professional soccer leagues, considering players’ participation in matches. Match running performance data were collected using the ChyronHego optical tracking system (TRACAB, New York, US). Soccer players were assigned to three groups based on their participation in matches: players who completed the full game (entire match players – EMP; *n* = 223,334 observations); players who participated for up to 85 minutes (replaced players – RP; *n* = 114,160 observations); and players who were substituted in during the second half (substitute players – SP; *n* = 43,700 observations). To calculate the 5-minute intervals for analysing temporal patterns in match running performance: i) total playing time was defined as the duration of the match, including injury time; ii) match data were segmented into pre-defined 5-minute intervals (i.e., 0–5, 5–10, 10–15 min, etc.); iii) extra time of the first and second halves were not considered in the analysis; the total playing time was only considered until 45 and 90 minutes from the first and second halves, respectively.

### Sample

A total of 820 matches of the 2022/23 season from the First (*n* = 367) and Second Spanish Division (*n* = 453) were considered for the analysis. Thus, 381,194 individual match observations from 1,251 professional soccer players were included. Goalkeepers were excluded from the analysis due to their unique role in the game. Data were provided by LaLiga, which informed all participants through its protocols. All data were anonymized in compliance with the Declaration of Helsinki to ensure the confidentiality of players and teams. The study received full approval from the Ethics Committee of the University of Extremadura; Vice-Rectorate of Research, Transfer and Innovation – Delegation of the Bioethics and Biosafety Commission (Protocol number: 239/2019).

### Variables and procedure

The optical tracking system TRACAB (ChyronHego VID, New York, NY) was employed to collect match running performance data. This multi-camera system comprises eight 4K High-Dynamic-Range cameras based on a positioning system (TRACAB—ChyronHego VTS), which records and analyses players’ X and Y positions from multiple angles, delivering real-time two-dimensional tracking at a sampling frequency of 25 Hz. Additionally, a customized report was generated using Mediacoach software (LaLiga, Madrid, Spain), which synchronized tracking data with video footage from each match. The validity and reliability of this system for the variables included in the analysis have been previously validated in the literature [19, 20]. Match running performance was divided into the following categories: total distance covered by players in metres (TD); very high-speed running distance (VHSR, 21–24 km × h^−1^); and sprinting speed running distance (Sprint, > 24 km × h^−1^). All physical variables were calculated as absolute distances in metres (m) during 5-minute intervals.

### Statistical analysis

All statistical analyses were conducted using RStudio [21]. Given the hierarchical structure of the sample—nested within groups and exhibiting a longitudinal design—linear mixed models (LMM) were deemed the most appropriate analytical approach.

LMM have proven effective in handling unbalanced and repeated-measures data [22, 23]. For example, match running performance variables are nested within players (i.e., each player has a record for every match they participated in, and each match contains observations from multiple players). Players, in turn, are nested within different teams across seasons. This cross-classified, multilevel structure makes LMM particularly suitable for analysing non-hierarchical data. Accordingly, a general multilevel modelling strategy was applied, incorporating fixed and random effects at different levels, as outlined by Heck and Thomas [22].

LMM were employed to examine the effects of time intervals on player activity. Initially, a two-level hierarchy was established for the analysis. Match running performance variables (e.g., distances covered at various speed thresholds) were treated as dependent variables, while time intervals (e.g., 0–5, 5–10, 10–15 min, etc.) and player types based on match participation (i.e., entire match, replaced, and substitutes) were included as fixed effects. The soccer player variable was incorporated as a random effect. Results were reported as coefficients with standard errors (coeff ± SE). Statistical significance was set at p < .05.

## RESULTS

**FIG. 1:**
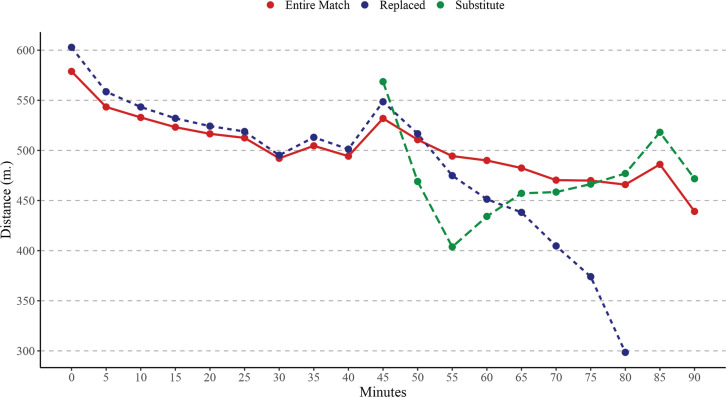
Evolution of Total Distance (TD) over 5-minute intervals time considering the match players participation. Note: m = metres.

**FIG. 2:**
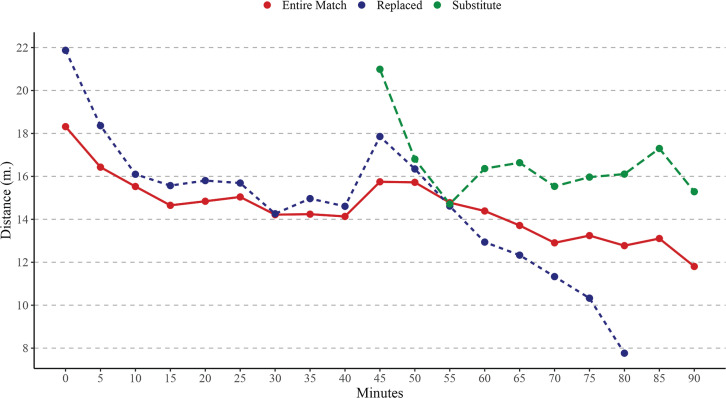
Evolution of Very high-speed running distance (VHSR) over 5-minute intervals time considering the match players participation. Note: m = metres.

**FIG. 3:**
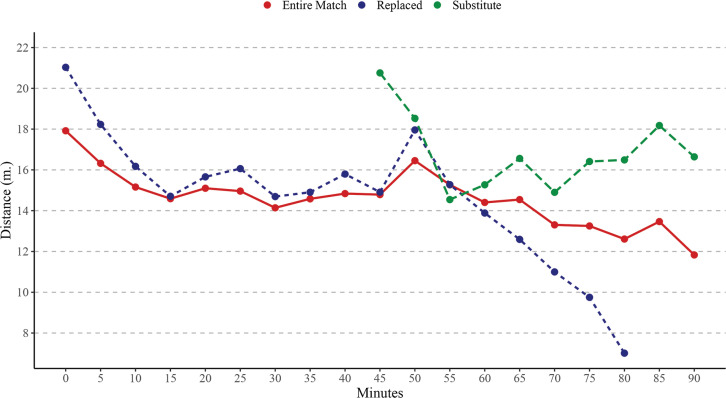
Evolution of Sprinting speed running distance (Sprint) over 5-minute intervals time considering the match players participation. Note: m = metres

Table 1 shows the differences in match running performance between time intervals based on the match players’ participation, whereas Figures 1, 2, and 3 depict the differences in match running performance over 5-minute time intervals. On the one hand, EMP covered significantly higher TD than RP in time periods 55–60 min (*p* < .001), 60–65 min (*p* < .001), 65–70 min (*p* < .001), 70–75 min (*p* < .001), 75–80 min (*p* < .001), 80–85 min (*p* < .001); and SP in time periods 50–55 min (*p* < .001), 55–60 min (*p* < .001), 60–65 min (*p* < .001), 65–70 min (*p* < .001), 70–75 min (*p* < .05). On the other hand, RP covered significantly higher TD than EMP in time periods 0–5 min (*p* < .001), 5–10 min (*p* < .001), 10–15 min (*p* < .05), 45–50 min (*p* < .001); and SP in time periods 50–55 min (*p* < .001), 55–60 min (*p* < .001), 60–65 min (*p* < .01). Finally, SP covered significantly higher TD than EMP in time periods 45–50 min (*p* < .001), 80–85 min (*p* < .01), 85–90 min (*p* < .001); and RP in time periods 65–70 min (*p* < .001), 70–75 min (*p* < .001), 75–80 min (*p* < .001), 80–85 min (*p* < .001).

**TABLE 1:** Match running performance differences during the 5-minute intervals considering the match players participation

Time periods (minutes)	TD (m)			*p*	VHSR (m)			*p*	Sprint (m)			*p*
		
	EMP	RP	SP		EMP	RP	SP		EMP	RP	SP	
		
	Coeff (SE)	Coeff (SE)	Coeff (SE)		Coeff (SE)	Coeff (SE)	Coeff (SE)		Coeff (SE)	Coeff (SE)	Coeff (SE)	
0–5	578.85 (3.78)	602.86 (3.90)		a ***	18.31 (.30)	21.87 (.32)		a ***	17.91 (.39)	21.03 (.42)		a ***

5–10	543.35 (3.78)	558.56 (3.90)		a ***	16.42 (.30)	18.36 (.32)		a ***	16.32 (.39)	18.22 (.42)		a **

10–15	532.91 (3.78)	543.34 (3.90)		a *	15.52 (.30)	16.09 (.32)		a ***	15.15 (.39)	16.17 (.42)	

15–20	523.20 (3.78)	532.02 (3.90)			14.65 (.30)	15.57 (.32)			14.58 (.39)	14.70 (.42)	

20–25	516.57 (3.78)	524.23 (3.90)			14.84 (.30)	15.80 (.32)			15.10 (.39)	15.65 (.42)	

25–30	512.54 (3.78)	518.88 (3.90)			15.04 (.30)	15.69 (.32)			14.96 (.39)	16.06 (.42)	

30–35	492.16 (3.78)	495.21 (3.90)			14.21 (.29)	14.25 (.32)			14.14 (.39)	14.69 (.42)	

35–40	504.60 (3.78)	513.08 (3.91)			14.23 (.29)	14.96 (.32)			14.58 (.39)	14.90 (.42)	

40–45	494.27 (3.78)	501.25 (3.91)			14.13 (.29)	14.60 (.32)			14.83 (.39)	15.79 (.42)	

45–50	531.90 (3.78)	548.54 (3.95)	568.62 (7.10)	a***, b***	15.74 (.29)	17.85 (.33)	20.98 (.85)	a***, b***	14.78 (.39)	14.90 (.43)	20.75 (1.09)	b***, c***

50–55	510.72 (3.78)	516.67 (3.95)	469.04 (6.58)	b***, c***	15.72 (.29)	16.34 (.33)	16.80 (.77)		16.45 (.39)	17.95 (.44)	18.52 (.99)	

55–60	494.35 (3.78)	474.86 (3.96)	403.75 (5.31)	a***, b***, c***	14.77 (.29)	14.61 (.33)	14.71 (.58)		15.26 (.39)	15.27 (.44)	15.54 (.74)	

60–65	489.98 (3.78)	451.33 (4.02)	434.14 (4.65)	a***, b***, c**	14.38 (.29)	12.93 (.35)	16.36 (.47)	a*, b*, c***	14.40 (.39)	13.88 (.45)	15.27 (.60)	

65–70	482.45 (3.78)	438.19 (4.13)	457.20 (4.35)	a***, b***, c***	13.71 (.29)	12.33 (.37)	16.63 (.41)	b***, c***	14.54 (.39)	12.59 (.48)	16.56 (.53)	a*, c***

70–75	470.35 (3.78)	404.71 (4.28)	458.46 (4.17)	a***, b*, c***	12.90 (.29)	11.33 (.40)	15.53 (.38)	b***, c***	13.30 (.39)	10.99 (.52)	14.90 (.49)	a**, c***

75–80	469.91 (3.78)	374.05 (4.54)	466.38 (4.04)	a***, c***	13.24 (.29)	10.32 (.44)	15.96 (.35)	a***, b***, c***	13.25 (.39)	9.75 (.58)	16.41 (.46)	a***, b***, c***

80–85	465.91 (3.78)	298.52 (5.09)	477.00 (3.95)	a***, b**, c***	12.77 (.29)	7.76 (.54)	16.10 (.33)	a***, b***, c***	12.60 (.39)	7.01 (.69)	16.48 (.44)	a***, b***, c***

85–90	486.08 (3.78)		518.09 (3.90)	b ***	13.10 (.29)	17.29 (.32)		b ***	13.46 (.39)		18.17 (.42)	b ***

*Notes*. Coeff = Coefficient; SE = Standard Error; m = metres; TD = Total distance; VHSR = Very high-speed running distance; Sprint = Sprint speed running distance; EMP = Entire match players; RP = Replaced players; SP = Substitute players;

a = differences between EMP and RP;

b = differences between EMP and SP;

c = differences between RP and SP;

* *p* < .05;

** *p* < .01;

*** *p* < .001.

Considering VHSR distance, EMP covered significantly higher VHSR than RP in time periods 60–65 min (*p* < .05), 75–80 min (*p* < .001), 80–85 min (*p* < .001). Moreover, RP covered significantly higher VHSR than EMP in time periods 0–5 min (*p* < .001), 5–10 min (*p* < .001), 10–15 min (*p* < .001), 45–50 min (*p* < .001). Finally, SP covered significantly higher VHSR than EMP in time periods 45–50 min (*p* < .001), 60–65 min (*p* > .05), 65–70 min (*p* < .001), 70–75 min (*p* < .001), 75–80 min (*p* < .001), 80–85 min (*p* < .001), 85–90 min (*p* < .001); and RP in time periods 60–65 min (*p* < .001), 65–70 min (*p* < .001), 70–75 min (*p* < .001), 75–80 min (*p* < .001), 80–85 min (*p* < .001).

Considering Sprint distance, EMP covered significantly higher Sprint than RP in time periods 65–70 min (*p* < .05), 70–75 min (*p* < .01), 75–80 min (*p* < .001), 80–85 min (*p* < .001). In addition, RP covered significantly higher Sprint than EMP in time periods 0–5 min (*p* < .001), 5–10 min (*p* < .01). Finally, SP covered significantly higher Sprint than EMP in time periods 45–50 min (*p* < .001), 75–80 min (*p* < .001), 80–85 min (*p* < .001), 85–90 min (*p* < .001); and RP in time periods 45–50 min (*p* < .001), 65–70 min (*p* < .001), 70–75 min (*p* < .001), 75–80 min (*p* < .001), 80–85 min (*p* < .001).

## DISCUSSION

The present study aimed to analyse differences in match running performance across 5-minute intervals, considering players’ match participation. To the best of our knowledge, this is the first study to examine the evolution of match running performance across 5-minute intervals while incorporating an analysis of players’ participation during matches in Spanish professional soccer leagues. Overall, the findings showed a decline in match running performance as match time progressed, with significant effects observed based on players’ roles. Specifically, EMP covered significantly greater TD than RP during intervals in the second half (from the 50^th^ minute onwards). Conversely, RP exhibited higher physical activity levels than EMP during intervals at the beginning of matches. Finally, SP covered significantly greater VHSR and Sprint distances compared to the other players during their time on the field.

Previous studies have shown that fatigue reduces professional soccer players’ performance by approximately 10% in TD between the first and second halves of a match and diminishes their ability to perform high-intensity efforts by 15–45% during the last 15 minutes [13, 24]. Additionally, effective playing time has been identified as a factor influencing the decline in match running performance, with TD being the most impacted variable, as reported by previous research [9, 10]. Our findings align with these observations, confirming a decrease in match running performance as the match progresses for players who complete the entire match, although these declines were not statistically significant. This could be attributed to advancements in regeneration techniques and individualized approaches to managing cumulative fatigue compared to earlier periods [13]. A recent study by Feng et al. [25], which analysed eight consecutive seasons of the Chinese Super League, reported no substantial changes in match running performance of soccer teams during the second half. Another plausible explanation for performance declines is the reduction in effective playing time in the second halves, as teams often use tactical interruptions to gain control of the game, thereby shortening effective playing time and reducing overall match running performance [9, 10]. Furthermore, our results revealed that match participation significantly influenced the evolution of match running performance during games. This suggests that each player role—whether completing the entire match, being replaced, or entering as a substitute—has distinct characteristics [26]. Lastly, it appears that with congested match schedules and the introduction of five substitutions, players’ physical activity and preparation requirements, depending on their roles, will evolve significantly in the future [27].

On the other hand, EMP covered significantly greater TD than RP during the second half, particularly from the 55^th^ to the 60^th^ minute onwards. Key players who are essential to the team’s performance typically play entire matches and are subjected to greater physical demands than other players throughout the season [28]. These players often adopt pacing strategies to maintain consistent physical activity or mitigate potential declines toward the end of matches [29]. For instance, after a particularly demanding phase of play, players may reduce their physical activity during subsequent intervals to restore energy reserves (e.g., muscle glycogen) [30]. Another commonly employed strategy by coaching staff is the rotation of starting players during key moments of the season, depending on their fatigue levels [31]. While both strategies are effective in minimizing activity declines—especially in relation to TD—it is evident that declines at higher running speeds cannot be completely avoided. This is particularly pronounced during the final 15 minutes of matches [30, 31].

A second key finding is that RP exhibited higher physical activity than EMP during the initial intervals of matches. This is likely because these players, who are substituted during the second half, are tasked with generating greater activity during the first half [32]. Our analysis clearly confirms that RP demonstrated higher activity levels across each 5-minute interval in the first half, particularly in TD, VHSR, and Sprint variables. Although these differences were not statistically significant in most cases, the trend was consistent across intervals, persisting up to the 55^th^ minute for TD and the 60^th^ minute for VHSR and Sprint. Beyond these points, a marked decline in these variables was observed with each subsequent 5-minute interval. This suggests that RP either expend maximum effort until their substitution or lack the preparation to sustain such activity levels for the full match, prompting their replacement by the coach [26]. Supporting this, Padrón-Cabo et al. [16] reported that most substitutions in LaLiga matches occur at half-time or between the 55^th^ and 85^th^ minutes of the second half.

Finally, SP covered significantly greater VHSR and Sprint distances compared to their counterparts during their time on the pitch. Interestingly, SP also exhibited lower TD performance than RP during the first 15 minutes of the second half. This finding suggests that the duration of time spent on the pitch likely influences match intensity [26]. Hills et al. [33] highlighted that substitutes are typically introduced at half-time or during the second half, often to mitigate fatigue effects and/or adjust tactics. The primary role of substitutes is to enhance team performance by providing a tactical advantage, particularly in offensive play. With their physical reserves, substitutes can stimulate the game by altering the scoreline and compensating for the overall performance drop within the team [34]. Mohr et al. [24] reported that elite substitutes introduced during the second half covered 25% more high-intensity running distance and 63% more sprinting distance than their counterparts. Subsequent research has consistently confirmed these patterns [14, 15], further underscoring the strategic importance of substitutions in modern soccer.

This study has several limitations that should be acknowledged. First, the analysis was limited to a single season, and technical activities were not considered. Furthermore, no internal load parameters, such as heart rate (HR) or rate of perceived exertion (RPE), were included. Future research should aim to incorporate the players’ positional data on the pitch. Identifying differences between playing positions, while accounting for 5-minute intervals and players’ participation, could offer deeper insights into teams’ pacing strategies. Additionally, comparing physical performance across league levels within countries or between international leagues represents a promising direction for further investigation.

## CONCLUSIONS

The present research has demonstrated that players’ participation in matches significantly impacts physical performance, particularly when analysed in 5-minute intervals. Overall, the results indicated a general decline in match running performance as the match progresses, with EMP, RP, and SP covering significantly greater VHSR and Sprint distances than other players during their respective periods of participation. These findings highlight the growing specialization in modern football based on players’ roles within the team. Furthermore, this study provides valuable insights into the evolution of match running performance over time.

In a practical approach, head coaches and strength and conditioning coaches should consider the participation of individual players and the planned minutes of play when preparing for matches. Moreover, assigning players an appropriate pacing strategy tailored to their role within the team during microcycle training—such as tapering or increasing high-intensity activity—can optimize their performance. Effective management of players’ match participation is a crucial strategy for maintaining or even enhancing the running performance of professional football teams, particularly during the second half of matches.

## Data Availability

Restrictions apply to the availability of these data. Data were obtained from LaLiga and are available at https://www.laliga.es/en with the permission of LaLiga.
